# Fuzzy logic: about the origins of fast ion dynamics in crystalline solids

**DOI:** 10.1098/rsta.2020.0434

**Published:** 2021-11-29

**Authors:** M. Gombotz, K. Hogrefe, R. Zettl, B. Gadermaier, H. Martin. R. Wilkening

**Affiliations:** ^1^ Institute for Chemistry and Technology of Materials, Christian Doppler Laboratory for Lithium Batteries, Graz University of Technology (NAWI Graz), Stremayrgasse, 9, 8010 Graz, Austria; ^2^ ALISTORE – European Research Institute, CNRS FR3104, Hub de l’Energie, Rue Baudelocque, 80039 Amiens, France

**Keywords:** nuclear magnetic resonance, solid electrolytes, diffusion, spins, relaxation

## Abstract

Nuclear magnetic resonance offers a wide range of tools to analyse ionic jump processes in crystalline and amorphous solids. Both high-resolution and time-domain ${\;}^{1,2}\text{H}$, ${\;}^{6,7}\text{Li}$, ${\;}^{19}\text{F}$, ${\;}^{23}\text{Na}$ NMR helps throw light on the origins of rapid self-diffusion in materials being relevant for energy storage. It is well accepted that ${\text{Li}}^{+}$ ions are subjected to extremely slow exchange processes in compounds with strong site preferences. The loss of this site preference may lead to rapid cation diffusion, as is also well known for glassy materials. Further examples that benefit from this effect include, e.g. cation-mixed, high-entropy fluorides ${\text{( Ba, Ca) F}}_{2}$, Li-bearing garnets (${\text{Li}}_{7}{\text{La}}_{3}{\text{Zr}}_{2}{\text{O}}_{12}$) and thiophosphates such as ${\text{LiTi}}_{2}{\left({\text{PS}}_{4}\right)}_{3}$. In non-equilibrium phases site disorder, polyhedra distortions, strain and the various types of defects will affect both the activation energy and the corresponding attempt frequencies. Whereas in $(\text{Me, Ca}){\text{F}}_{2}$ (Me=Ba, Pb) *cation* mixing influences F anion dynamics, in ${\text{Li}}_{6}{\text{PS}}_{5}\text{X}$ (X=Br, Cl, I) the potential landscape can be manipulated by *anion* site disorder. On the other hand, in the mixed conductor ${\text{Li}}_{4+x}{\text{Ti}}_{5}{\text{O}}_{12}$ cation-cation repulsions immediately lead to a boost in ${\text{Li}}^{+}$ diffusivity at the early stages of chemical lithiation. Finally, rapid diffusion is also expected for materials that are able to guide the ions along (macroscopic) pathways with confined (or low-dimensional) dimensions, as is the case in layer-structured ${\text{RbSn}}_{2}{\text{F}}_{5}$ or ${\text{MeSnF}}_{4}$. Diffusion on fractal systems complements this type of diffusion.

This article is part of the Theo Murphy meeting issue ‘Understanding fast-ion conduction in solid electrolytes’.

## 1. Introduction

Powerful solid-state ionic conductors are needed to develop advanced sensors and energy storage systems such as rechargeable Li-ion and Na-ion batteries. To realize such devices materials with specific ionic conductivities reaching or even exceeding $1\;{\text{mS cm}}^{-1}$ at room temperature are needed.

**Figure 1.**
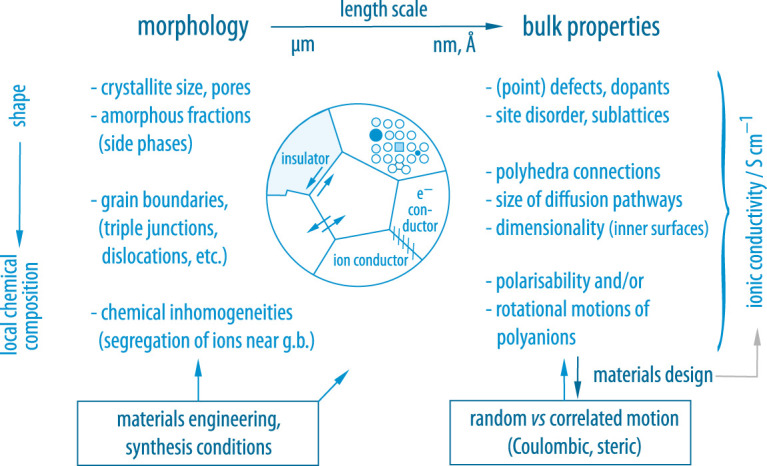
Length-scale-dependent adjusting screws to tune ion dynamics in condensed matter. While materials engineering and synthesis conditions will affect macroscopic morphology such as the nature of grain boundaries in polycrystalline samples, bulk dynamic properties are to be manipulated by a clever choice of, for example, dopants, anions and crystal structures that offer, in particular, spatially confined diffusion pathways being able to guide the ions over long distances. Moreover, synthesis conditions may also influence local structures. (Online version in colour.)

**Figure 2.**
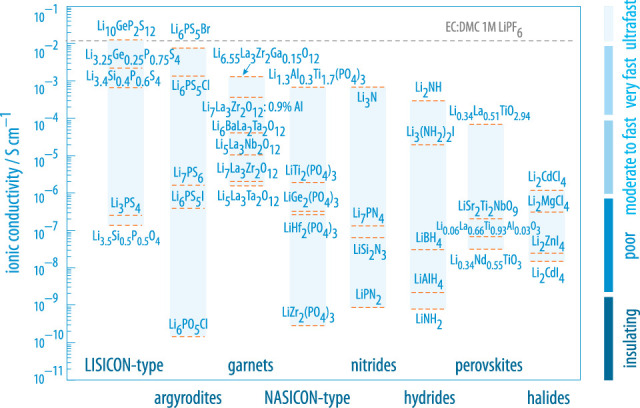
Selected classes of materials and corresponding examples of solid electrolytes with ion conductivities ranging from very poor values to values in the $10\;{\text{mS cm}}^{-1}$ regime, adapted from [1]. This short overview does raise no claim to completeness as, especially in recent years, further materials were characterized such as ${\text{Li}}_{3}{\text{MX}}_{6}$ (M=Y, In, La; X=Cl, Br) [6–12] and variants of the Li-containing argyrodites [13]. (Online version in colour.)

Over the last couple of years, many promising oxides, phosphates, thiophosphates and hydrides were introduced that meet this important requirement [1–3]. Some of them, however, fail to fulfill other important properties such as electrochemical stability, mechanical robustness or low interfacial (contact) resistances when in close contact with materials such as metallic Li or Na [4]. Therefore, we need to understand the driving forces that lead to fast ion conduction to turn other compounds, already fulfilling some of the technological demands, into suitable ceramic electrolytes. Independent of having any applications in mind, understanding ion transport or self-diffusion in solids is driven by academic curiosity. Since the discovery of fast Na ion dynamics in layer-structured beta’-alumina, the irregular diffusion of ions in both crystalline and amorphous solids has become a vital topic in materials science [5]. It depends on many different factors as is illustrated in figure 1. In figure 2, an overview is given showing the ionic conductivities of selected compounds from different classes of materials [1]. The thiophosphate ${\text{Li}}_{10}{\text{GeP}}_{2}{\text{S}}_{12}$ [14] and its relatives [15], as well as the class ceramic ${\text{Li}}_{7}{\text{P}}_{3}{\text{S}}_{11}$ [16–18] (${\text{Li}}_{2}{\text{S : P}}_{2}{\text{S}}_{5}$ [19]), and argyrodite-type ${\text{Li}}_{6}{\text{PS}}_{5}\text{Br}$ [20,21] definitely belong to the frontrunners of materials with conductivities in some cases lying in the order of $10\;{\text{mS cm}}^{-1}$ (at room temperature, RT). Less soft materials which are envisaged to be used in ceramic batteries include garnet-type compounds based on ${\text{Li}}_{7}{\text{La}}_{3}{\text{Zr}}_{2}{\text{O}}_{12}$ (LLZO) [22,23] and materials crystallizing with the well-known NASICON (Na superionic conductor) structure, such as lithium aluminium titanium phosphate (LATP, ${\text{Li}}_{1.3}{\text{Al}}_{0.3}{\text{Ti}}_{1.7}{\left({\text{PO}}_{4}\right)}_{3}$) [24] or compounds with similar composition [25].

As mentioned above, the origins behind fast ion conduction are, however, manifold and not always easy to grasp. It resembles the situation of fuzzy logic [26] as a range of properties may affect overall ionic transport and the elementary steps of ion hopping. One may generally ask whether a single property is mainly responsible for cation or anion dynamics in a given compound or whether a couple of properties, possibly influencing each other, govern overall dynamics. Figure 1 differentiates between macroscopic, that is, morphology related properties of a sample, and microscopic, bulk properties. Especially for oxide materials, we often witness relatively fast bulk ion dynamics that is, however, blocked by highly resistive grain boundary regions. As has been shown for many examples, the synthesis conditions can sensitively affect a range of morphological properties such as crystallite and pore sizes, amorphous fractions, the nature of grain boundaries and the occurrence of chemical inhomogeneities.

Bulk properties can mainly be adjusted by the smart introduction of defects, structural (site) disorder or stress. The latter can be introduced by mechanical treatment, for example [27]. The use of mechanical energy to prepare complex compounds is called mechanosynthesis; this kind of synthesis often yields high-pressure phases or non-equilibrium (metastable) compounds that may show considerably different properties when compared with their structurally relaxed but chemically identical counterparts [27–30]. Moreover, modifying the polyanion lattice by replacing the original anions with ions differing in size and polarisability [31] may affect the bottlenecks of diffusion pathways, bonding situations and electrostatic interactions. The latter factors often determine whether correlated or uncorrelated motion predominates. Correlated motion is expected for strong cation-cation or cation-anion interactions, which also includes concerted or collective motions. Moreover, the coupling of translational and rotational dynamics will result in correlated motion. It leaves characteristic signatures in both conductivity isotherms and time-domain nuclear magnetic resonance (NMR) experiments [32]. Similar features are also seen for dimensionality effects including also fractals; in many cases, spatial confinement is attractive for fast cation and anion diffusion as the ions are guided along the inner or buried interfaces of such compounds, see, e.g. F anion dynamics in ${\text{MeSnF}}_{4}$ (Me=Ba, Pb) [27]. Of course, whereas fast two-dimensional diffusion [33] is seen in layered materials, this principle does not work for channel-structured materials, whose one-dimensional pathways may easily get blocked by foreign atoms or by accumulated ions. An exception is given for ${\text{Li}}_{7}{\text{Si}}_{12}$ where Li cations are guided along the stacked ${\text{Si}}_{5}$ rings [34].

Besides these adjusting screws to manipulate macroscopic and microscopic ion dynamics, we have the possibility to take advantage of interfacial effects [35] in both single-phase and two-phase conductor:insulator or ion-conductor:electron-conductor composites [27,36–38]. If the crystallite regions are reduced to nanometer-sized dimensions, a network of percolating diffusion pathways can be generated, which in the case of ${\text{Li}}_{2}\text{O}:{\text{Al}}_{2}{\text{O}}_{3}$ or $\text{LiF}:{\text{Al}}_{2}{\text{O}}_{3}$ resulted in fast ion dynamics [39,40]. The most famous example are nm-thick heterolayers of ${\text{BaF}}_{2}$ and ${\text{CaF}}_{2}$ that benefit from non-trivial space charge effects showing up at the interfacial regions [41].

The manifold possibilities to influence ion dynamics are directed toward a single goal: decreasing (local) activation barriers $E_{\text{a}}$, that is, flattening the potential landscape, and increasing the so-called attempt frequency $1/\tau_{0}$ that also governs the cation (or anion) jump rate, for which, in the easiest case, a strict Arrhenius behaviour is assumed: $1/\tau =1/\tau_{0}\exp(-E_{\text{a}}/(k_{\text{B}}T))$. Here, T denotes the absolute temperature and $k_{\text{B}}$ is Boltzmann’s constant. 1/τ is directly proportional to the ionic mobility μ that governs ionic conductivity σ according to σ=qNμ; q denotes the charge of the mobile species and N represents the charge carrier density.

In the following, we will present selected case studies of our own laboratory to highlight some of the main strategies to improve cation and anion self-diffusivity. Most of the compounds included here were studied with the help of time-domain NMR techniques, that is, diffusion-controlled spin-lattice relaxation (SLR) measurements [32,42]. Such measurements are extremely sensitive to motion-induced spin fluctuations; depending on the temperature and the frequencies used to sample the corresponding SLR rates, NMR gives access to both short-range and long-range dynamic parameters [32]. It is a contactless method: hence, no post-preparation steps, as in the case of other methods, are necessary to prepare the powdered samples.

## 2. Case studies

### (a) Lithium pentatitanate, the zero-strain anode material for batteries

**Figure 3.**
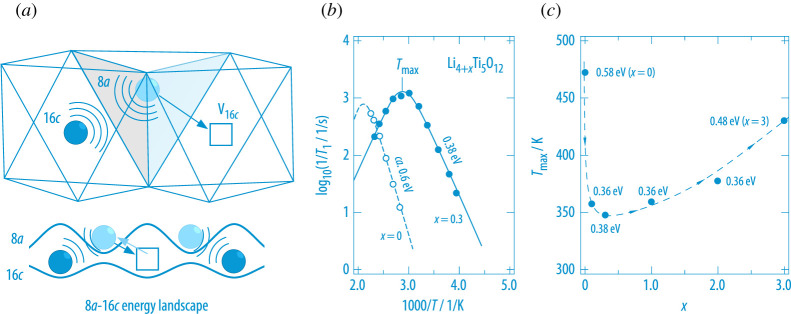
(*a*) Frustrated situation in LTO: For x>0, the Li-ions occupy two different sublattices, whose polyhedra share common faces; this situation results in fast Li hopping as the ions do not find a suitable void to reside [43,44]. The ions simply cannot escape from repulsive interactions as each vacant 16*c* site ($V_{16c}$) is connected to occupied 8*a* sites. This situation resembles somehow that which is usually met in glassy ion conductors. (*b*) ${\;}^{7}\text{Li}$ NMR spin-lock rate peaks of LTO with x=0 and x=0.3. (*c*) Change of $T_{max}$ with composition *x*; the activation energies are also included. (Online version in colour.)

Direct Li-Li Coulomb interactions generated when the Li-ions start to occupy the originally empty Li sublattice lead to fast ${\text{Li}}^{+}$ dynamics in ${\text{Li}}_{4+x}{\text{Ti}}_{5}{\text{O}}_{12}$ (LTO), with x being slightly larger than 0 [43,44]. LTO crystallises with spinel structure (see figure 3) and the Li-ions occupy two distinct crystallographic sites, they share the 16d site with ${\text{Ti}}^{4+}$ and occupy all tetrahedral 8a sites. As has been shown by high-resolution ${\;}^{6}\text{Li}$ 2D exchange NMR the Li-ions on 16d do not take part in fast ion transport [44]. The chemical insertion of additional Li (${\text{Li}}^{+}$, ${\text{e}}^{-}$) changes the oxidation state of Ti from +4 to +3 and forms a mixed-conducting compound. The new Li-ions enter the structure by occupying the empty 16c octahedral sites that are connected to 8a by face sharing, see figure 3. Occupying the originally empty 16c sublattice immediately causes local 16*c*–8*a* Li-Li interactions forcing the 8a Li-ion to leave its site. This repulsive interaction leads to a redistribution of the ${\text{Li}}^{+}$ ions. Recently, this kind of kick-out mechanism has also been identified as the relevant one by theory [45]. Importantly, partial filling of the two sublattices (8*a* and 16*c*), having two different energy landscapes, leaves behind a frustrated situation for ${\text{Li}}^{+}$ as Li-Li repulsive interactions cannot be eliminated any longer. This situation results in a steep increase of ${\text{Li}}^{+}$ self-diffusivity involving the 8*a*-16*c*-8*a* pathway.

${\;}^{7}\text{Li}$ NMR SLR, carried out in the so-called rotating-frame of reference, turned out to be a perfect tool to probe this drastic change in Li-ion dynamics [43]. In contrast to ordinary $1/T_{1}\;\text{NMR}$, the spin-lock rates $1/T_{1\varrho }$ are less affected by Curie-Weiss behaviour in ${\text{Li}}_{4+x}{\text{Ti}}_{5}{\text{O}}_{12}$ as the contribution through diffusive motions with motional correlation rates in the order of $10^{5}\;{\text{s}}^{-1}$ governs the rates [43]. For x=0, the temperature-dependent SLR rates $1/T_{1\varrho }$ pass through a classic peak at relatively high T if plotted as $\log_{\text{10}}(1/T_{1\varrho })$ versus 1/T. The slight increase of x from 0 to 0.3 shifts the rate peak toward much lower temperatures and reduces the NMR activation energy from *ca* 0.60 eV to 0.38 eV. In general, such peaks arise at those temperatures $T_{\text{max}}$ at which the mean motional correlation rate $1/\tau_{c}\approx 1/\tau$ reaches the order of the angular frequency used to probe the SLR rates. Here, this frequency, which is the so-called locking frequency, was 20 kHz. Thus, at $T_{\text{max}}=330\;\text{K}$ the average jump rate is in the order of $10^{5}\;{\text{s}}^{-1}$ for x=0.3. For comparison, this rate is reached for x=0 only at much higher $T_{\text{max}}$. The change of $T_{\text{max}}$ over the whole compositional range x is illustrated in figure 3. At sufficiently high values of x the activating energy increases again; note that at x=3 all 8a sites are empty and the 16c sublattice is completely filled with Li-ions resulting in rocksalt-type LTO.

**Figure 4.**
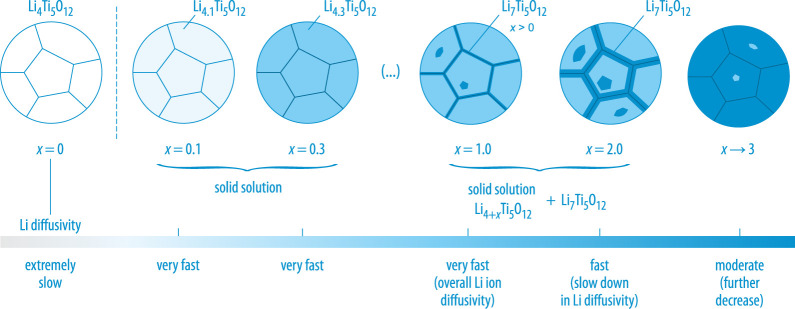
The lithiation process of polycrystalline ${Li}_{4}{\text{Ti}}_{5}{\text{O}}_{12}$ with the grain boundary regions indicated by thin solid lines. At very low *x* values, chemical lithiation turns LTO into a solid solution with fast ${\text{Li}}^{+}$ diffusivity [44]; with proceeding lithiation a two-phase system might evolve that shows ${\text{Li}}_{7}{\text{Ti}}_{5}{\text{O}}_{12}$ embedded in the ${\text{Li}}_{4+x}{\text{Ti}}_{5}{\text{O}}_{12}$ (x>0) matrix. The formation of ${\text{Li}}_{7}{\text{Ti}}_{5}{\text{O}}_{12}$ is expected to occur at the grain boundary regions The overall self-diffusivity decreases when the level of full lithiation is reached at x=3. As suggested by Wagemaker and co-workers a subnanometer phase morphology might also be involved to explain the resulting flat insertion potential of LTO [46]. (Online version in colour.)

The steep increase in Li diffusivity as is seen by ${\;}^{7}\text{Li}$ spin-lock NMR—which, incidentally, affects all Li spins in ${\text{Li}}_{4.3}{\text{Ti}}_{5}{\text{O}}_{12}$—can be used to draft a picture on the phases being involved in LTO when used as an ‘insertion’ host. At low values of x, the poorly conducting and electrically insulating LTO immediately turns into a mixed-conducting solid-solution with fast ${\text{Li}}^{+}$ diffusivity, which was also verified by high-resolution ${\;}^{6}\text{Li}$ MAS NMR [44]. With increasing x the formation of a two-phase system is expected that is composed of ${\text{Li}}_{4+x}{\text{Ti}}_{5}{\text{O}}_{12}$ (with x>0, phase 1) and the less conducting rocksalt-type ${\text{Li}}_{7}{\text{Ti}}_{5}{\text{O}}_{12}$ phase, see figure 4 [44]. A two-phase picture is usually used to explain the extremely flat (de-)insertion plateau of LTO in electrochemistry. It is important to note that the nature of the two-phase system formed electrochemically is expected to depend on the exact lithiation kinetics [47], also involving internal interfacial properties [46], which can be controlled by both temperature and the charge-discharge rates chosen. Fast Li diffusion in a subnanometer (interfacial) phase morphology seems to be important to explain the excellent rate performance of macroscopically two-phase LTO that is seen at sufficiently high x values [45,46].

### (b) The loss of site preferences in oxides and thiophosphates

**Figure 5.**
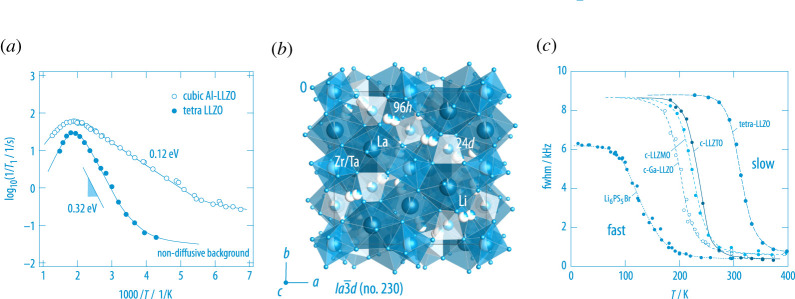
(*a*) ${\;}^{7}\text{Li}$ NMR spin-lattice relaxation rates of garnet-type ${\text{Li}}_{7}{\text{La}}_{3}{\text{Zr}}_{2}{\text{O}}_{12}$; here the response of the tetragonal form is compared with that of the cubic one, which is stabilized by Al incorporation [48,49,53]. (*b*) Crystal structure of cubic ${\text{Li}}_{7}{\text{La}}_{3}{\text{ZrTaO}}_{12}$ which shows a similar ionic conductivity as the Al-stabilized form. The sites 24*d* and 96*h* form a three-dimensional network that enables the ions to move fast through the rigid crystal structure. (*c*) Comparison of the ${\;}^{7}\text{Li}$ NMR motional curve (MN) of tetra-LLZO with those of various cubic variants. The earlier line narrowing sets in (on the temperature scale), the faster the ion hopping processes that average dipole-dipole (Li-Li) couplings. For comparison, the MN curve of a thiophosphate ${\text{Li}}_{6}{\text{PS}}_{5}\text{Br}$ is also included [54]. (Online version in colour.)

Materials with a large number of vacancies and many available Li voids, which can easily be reached by the charge carriers, also include oxides, such as Al-stabilized ${\text{Li}}_{7}{\text{La}}_{3}{\text{Zr}}_{2}{\text{O}}_{12}$ [48,49], and thiophosphates such as ${\text{LiTi}}_{2}{\left({\text{PS}}_{4}\right)}_{3}$ [50]. In contrast to compounds with strong site preferences such as ${\text{LiAlO}}_{2}$ [51] and ${\text{Li}}_{2}{\text{ZrO}}_{3}$ [52], the Li sites of the partially occupied sublattices in these compounds are connected by small activation energies facilitating rapid local and also effective through-going ion transport. Whereas for the tetragonal form of LLZO the Li-ions are distributed over the Li voids in a regular manner, in cubic LLZO, which is stabilized by replacing some of the Li-ions by Al ions, the sites 24*d* and the split-site 96*h* are only partially occupied. These sites form a three-dimensional network of fast diffusion pathways including the pathway 24*d*-96*h*-24*d* and also hopping between crystallographic similar sites, see figure 5. Fast ion exchange in cubic-LLZO overcompensates any blocking effect through the ${\text{Al}}^{3+}$ ions that likely occupy sites originally filled by ${\text{Li}}^{+}$. The effect of partially occupied sublattices on Li-ion dynamics and, thus, on the ${\;}^{7}\text{Li}$ NMR SLR rates $1/T_{1}$ is clearly seen in figure 5. While for ordered tetra-LLZO an activation energy on the low-T side of the rate peak of 0.32 eV is obtained [53], in cubic Al-LLZO the local jump processes are to be characterized by a barrier as low as 0.12 eV [48,49]. This value, which is associated with the asymmetry of the whole rate peak, points to highly correlated ion dynamics in Al-LLZO. Long-range ion transport in the two compounds, as is seen by spin-lock NMR, is governed by activation energies of 0.52 eV and 0.35 eV [48,49,53]. The fact that the rate peaks appear at almost the same $T_{\text{max}}$ shows that the pre-factor of the corresponding Arrhenius relation of Al-LLZO is much lower than that in the structurally ordered form of ${\text{Li}}_{7}{\text{La}}_{3}{\text{Zr}}_{2}{\text{O}}_{12}$ [54].

In figure 5, we also compare the so-called ${\;}^{7}\text{Li}$ NMR motional narrowing curve of tetragonal LLZO with those of some of its cubic variants for which Zr is replaced by Ta or Mo [54]. Ga-stabilized LLZO crystallizes in an acentric cubic modification of LLZO and shows even higher ionic conductivities because an additional Li pathway is generated in this structure as has been seen by spin-lock ${\;}^{7}\text{Li}$ SLR NMR [55]. In general, NMR motional narrowing shows up when the mean Li jump rate reaches the order of the spectral width of the NMR line. The width of the latter is given by the Li-Li dipolar interactions according to the Van Vleck formalism. With increasing diffusivity dipolar interactions are increasingly averaged resulting in a narrowed NMR line. The more the curve is shifted toward lower T, the more rapid Li-ion dynamics takes place. Models introduced by Abragam as well as by Hendrickson and Bray have been introduced to analyse such curves and to extract activation energies [56]. The temperature-independent region at low T is often called the rigid-lattice regime in which 1/τ is much lower than the spectral width.

**Figure 6.**
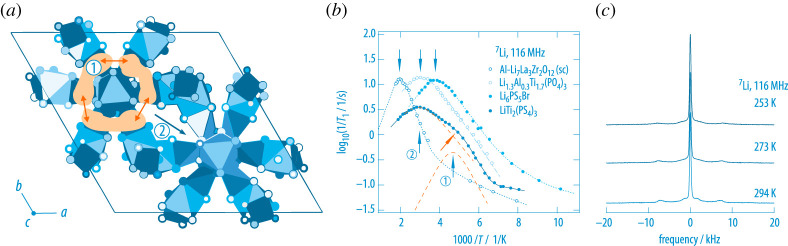
(*a*) Crystal structure of ${\text{LiTi}}_{2}{\left({\text{PS}}_{4}\right)}_{3}$ (LTPS) offering at least two dynamic modes: an intra-ring diffusion process and an inter-ring process that enables long-range ion transport. (*b*) The corresponding ${\;}^{7}\text{Li}$ NMR relaxation rates in comparison with diffusion-induced rates from other ion conductors. In the case of ${\text{LiTi}}_{2}{\left({\text{PS}}_{4}\right)}_{3}$, two maxima are passed through by $1/T_{1}$ which we attribute to the inter- (2) and intra-cage (1) ion dynamics [50]. The latter rate peak even exceeds that of ‘superionic’ ${\text{Li}}_{6}{\text{PS}}_{5}\text{Br}$ that also shows fast intra-cage ion dynamics (see below). Dashed lines show a deconvolution of the overall response with two BPP-type peaks [57]. Arrows point to the peak maxima. (*c*) ${\;}^{7}\text{Li}$ NMR spectra revealing that multiple jump processes determine ion dynamics in LTPS as the quadrupole powder pattern changes (and sharpens) several times with increasing temperature. At T=294 K,  the (almost) final pattern is observed that includes all relevant averaging processes with respect to the various electric quadrupole interactions. (Online version in colour.)

The thiophosphate ${\text{LiTi}}_{2}{\left({\text{PS}}_{4}\right)}_{3}$ (LTPS) represents one of the unique examples that provides several Li sites embedded in a very flat potential landscape [50]. Thus, geometric frustration is at play to explain the rapid exchange of Li-ions among the various rather broad Li voids. Indeed, pulsed field gradient NMR and diffusion-induced SLR point to rather fast Li-ion exchange processes [50]. Importantly, ${\;}^{7}\text{Li}$ NMR senses two different motional processes. The Li-ions are able to perform extremely fast within-site motions and can jump within a ring structure from site to site. The corresponding residence time of this *intra*-ring ion process, see figure 6, is in the order of 1 ns at 200 K [50]. Jumps *between* the rings are to be characterized by the second NMR rate peak (2) that appears at 330 K, see figure 6 which shows the deconvolution of the overall ${\;}^{7}\text{Li}$ NMR SLR response with two rate peaks according to the model for isotropic diffusion introduced by Bloembergen, Purcell and Pound (BPP) [57]. ${\;}^{7}\text{Li}$ NMR line shape measurements reveal that a range of different electric quadrupolar interactions are averaged through ionic motions when analysing the corresponding powder patterns of these NMR spectra as a function of temperature. A final (motionally averaged) quadrupole powder pattern is obtained only well above ambient temperature pointing to a distribution of fast and slow diffusion processes governing overall ion dynamics in LTPS.

### (c) Cation disorder influencing anion dynamics and vice versa

**Figure 7.**
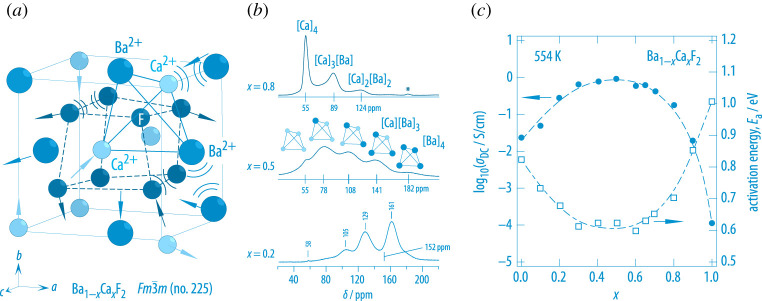
(*a*) Mechanical mixing forces the fluorides ${\text{BaF}}_{2}$ and ${\text{CaF}}_{2}$ to form a metastable, non-equilibrium solid-solution ${\text{Ba}}_{1-x}{\text{Ca}}_{x}{\text{F}}_{2}$ (0<x<1); strain and stress due to the significantly differing ionic radii causes the F anions to diffusive in a highly irregular potential landscape. (*b*) Mixing at atomic scale can be verified by high-resolution ${\;}^{19}\text{F}$ MAS NMR spectroscopy at spinning speeds of up to 60 kHz. Each signal reflects a distinct ${\text{[Ba]}}_{n}{\text{[Ca]}}_{4-n}$ environment sensed by the spin-1/2 F nuclei [58]. (*c*) At x=0.5, being the compound with highest possible disorder, the ion conductivity $\sigma_{DC}$ of the mixture passes through its maximum, the corresponding activation energy $E_{a}$ is minimal for these intermediate compositions [58]. (Online version in colour.)

Frustrated (or highly heterogeneous) potential landscapes generated by site disorder are also at the origin of fast anion dynamics in the earth-alkaline system $(\text{Ba, Ca}){\text{F}}_{2}$ [58,59]. While ${\text{BaF}}_{2}$ and especially ${\text{CaF}}_{2}$ are to be regarded as poor anion conductors (see figure 7*c*), the mechanically forced formation of the nanocrystalline solid-solution $(\text{Ba},\;\text{Ca}){\text{F}}_{2}$ has to be characterized by significantly enhanced anion dynamics [60,61]. As $(\text{Ba, Ca}){\text{F}}_{2}$ is sensitive to heat treatment at temperatures of 700 K, it cannot be prepared by conventional solid-state synthesis. Joint high-energy ball milling of the binary starting materials ${\text{BaF}}_{2}$ and ${\text{CaF}}_{2}$ does, however, yield a metastable solid-solution ${\text{Ba}}_{1-x}{\text{Ca}}_{x}{\text{F}}_{2}$ (0<x<1) that offers rapid F anion exchange processes greatly exceeding those being present in nanocrystalline ${\text{BaF}}_{2}$, which crystallizes with fluorite structure (space group $Fm\bar{3}m$). Note that although ionic conductivity in ${\text{nano-BaF}}_{2}$ exceeds that of micro- or single crystalline ${\text{BaF}}_{2}$, it does not reach the conductivity range of the cation-mixed solid solution [61,62].

As illustrated in figure 7, site disorder of the two earth-alkaline cations showing mismatch in ionic radius ($r({\text{Ba}}^{2+})>r({\text{Ca}}^{2+}))$ is expected to cause steric as well as Coulombic interactions severely distorting the fluorite lattice. F anion site frustration finally results in a decrease of the overall (migration) activation energy $E_{a}$ and an increase of the ionic (direct current, DC) conductivity $\sigma_{\text{DC}}$ by two orders of magnitude when comparing $\sigma_{\text{DC}}$ of ${\text{Ba}}_{1-x}{\text{Ca}}_{x}{\text{F}}_{2}$ (x=0.5) with that of non-substituted nanocrystalline ${\text{BaF}}_{2}$ (x=0) [58]. Local ion dynamics and the change of the corresponding Arrhenius pre-factor, which is highest for heavily mixed samples, were also studied by NMR relaxometry, quite recently [62]. It turned out that if studied from an atomic-scale point of view increased ion dynamics at intermediate values of composition is reflected by increased absolute diffusion-induced ${\;}^{19}\text{F}$ NMR SLR rates rather than by a distinct minimum in activation energy. Hence, the pre-factor of the underlying Arrhenius relation that governs the motional correlation rate $1/\tau_{c}$, which is determined by the attempt frequency and entropy effects, is identified as the parameter that directly enhances short-range ion dynamics in metastable ${\text{Ba}}_{1-x}{\text{Ca}}_{x}{\text{F}}_{2}$ [62]. The pre-factor of the corresponding diffusion coefficient also depends on the exact diffusion mechanism, which is in many cases not known. In general, the various mechanisms may involve vacancies, interstitial sites and trapping effects that can limit overall diffusion. For ${\text{Ba}}_{1-x}{\text{Ca}}_{x}{\text{F}}_{2}$, concerted ion migration, which is expected for LLZO and argyrodite-type materials (see above), seems to also play an important role to explain the anomalies seen in NMR SLR measurements [62].

${\;}^{19}\text{F}$ magic angle spinning (MAS) NMR has been used to verify mixing of the earth-alkaline cations at atomic scale and to exclude extensive segregation or the formation of Ba-rich and Ca-rich clusters [58,62]. As can be seen in figure 7*b*, the spectrum belonging to x=0.5 is composed of five distinct signals that mirror the possible ${\text{[Ba]}}_{n}{\text{[Ca]}}_{4-n}$ (n=0,1,2,3,4) next-neighbour (tetrahedral) environments sensed by the spin-1/2 nucleus ${\;}^{19}\text{F}$. For comparison, the isotropic NMR lines of ${\text{BaF}}_{2}$ and ${\text{CaF}}_{2}$ appear at 58 ppm and 152 ppm, respectively.

**Figure 8.**
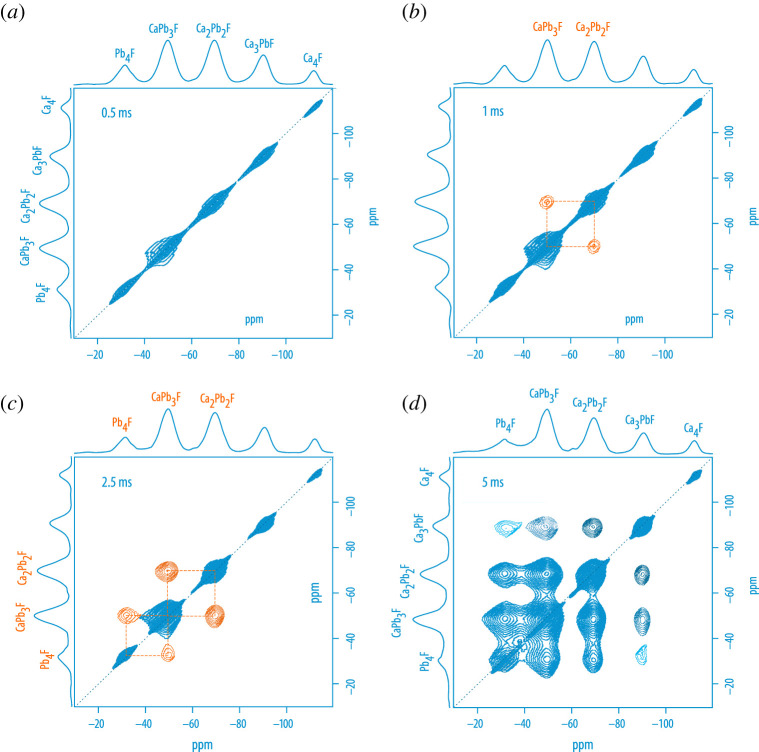
(*a*–*d*) Contour plots of selected mixing-time dependent ${\;}^{19}\text{F}$ 2D exchange MAS NMR spectra of mechanosynthesized ${\text{Pb}}_{0.6}{\text{Ca}}_{0.4}{\text{F}}_{2}$ [63]. Mixing times were varied from 0.5 ms to 5.0 ms; F anion exchange, as is verified by off-diagonal intensities (in orange), is energetically preferred between site-disordered environments rather than between Pb-rich or Ca-rich F sites, see (*b*) and (*c*). As in ${\text{Ba}}_{1-x}{\text{Ca}}_{x}{\text{F}}_{2}$, the first tetrahedral coordination shell of F is formed by four cations finally resulting in five NMR lines, see the spectra in the F1 and F2 directions. At a mixing time of 5 ms exchange is seen between most sites except the Ca-rich ones where cross peaks are of very low intensity (not seen here). This observation is reasonable as ${\text{CaF}}_{2}$ is a much poorer ionic conductor as ${\text{PbF}}_{2}$ is. However, we cannot exclude that at too large mixing times the cross peaks in (*d*) are already affected by spin-diffusion effects. (Online version in colour.)

The analogous (non-equilibrium) system ${\text{Pb}}_{1-x}{\text{Ca}}_{x}{\text{F}}_{2}$ was used to visualize the F anion hopping preferences by means of ${\;}^{19}\text{F}$ 2D exchange MAS NMR [63]. Contour plots of such experiments, carried out at short mixing times to exclude the influence of spin-diffusion effects, are shown in figure 8. NMR reveals that hopping between structurally similar sites is energetically preferred by the jumping ions. F ion exchange predominantly involves the Pb-rich F sites that are distorted by the smaller Ca ions. Conductivity measurements confirm enhanced dynamics in ${\text{Pb}}_{1-x}{\text{Ca}}_{x}{\text{F}}_{2}$, which finally leads to improved long-range ion transport in the cation-mixed system.

The preference of ions to hop between similar sites is evocative of the explanation that was used to explain the sharp decrease in conductivity in glasses with two mobile charge carriers, such as Li and Na. Such glasses show a typical mixed alkali effect [64–66], i.e. the activation energy, as a function of composition, passes through a maximum while the conductivity, which drops immediately as the second mobile species is introduced into the glass structure, passes through a minimum. Such memory effects of site occupancy were supported by theory and extended X-ray absorption fine structure measurements [66,67].

Similar effects, that is, taking benefit from cation mismatch, are used to enhance F anion transport in tysonite-type materials such as ${\text{La}}_{1-x}{\text{Ba}}_{x}{\text{F}}_{3-x}$ [68]. The same holds for heterovalent replacement of ${\text{La}}^{3+}$ by ${\text{Sr}}^{2+}$, for which a decrease of the activation energy from 0.75 eV (${\text{nano-LaF}}_{3}$, x=0) to 0.49 eV (x=0.1) is observed by DC conductivity measurements [69]. For the latter material, the trend of the activation energies and the pre-factors $\sigma_{0}(x)$ of the conductivity Arrhenius lines is in agreement with the so-called Meyer-Neldel rule [70], which describes an enthalpy-entropy compensation effect. This compensation effect is based on a linear dependence of the pre-factor on the activation enthalpy (or energy). The higher the activation enthalpy, the higher the pre-factor, which is affected by entropy contributions, attempt frequencies and, in the case of ionic conductivity, also by the effective number of charge carriers. Recently, a similar trend has also been found for LLZO-type conductors [71] and other compounds such as thiophosphates [31,72]. The semi-empirical relationship seems to be valid at least for the averaged, macroscopic ion transport. Probing diffusion properties, preferably over a large length scale is, however, needed to understand ion dynamics in detail.

**Figure 9.**
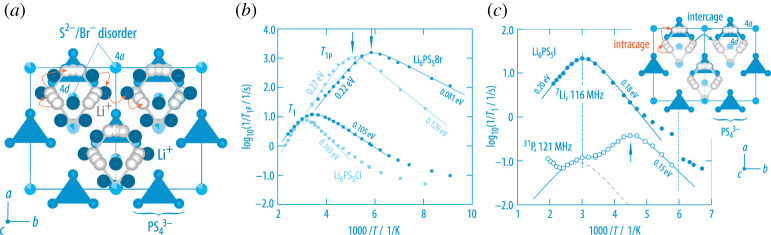
(*a*) Crystal structure of anion disordered argyrodite-type ${\text{Li}}_{6}{\text{PS}}_{5}\text{X}$ (X=Br); while Br and S are distributed among different crystallographic positions, the ${\text{Li}}^{+}$ ions do occupy several positions forming cages (grey spheres) and further sites (dark blue spheres) connecting these. This situation contrasts that which is seen for ${\text{Li}}_{6}{\text{PS}}_{5}\text{I}$ where S and I anions form ordered sub-lattices with I not occupying the position 4*d* inside the Li-cages, see inset of the figure shown on *c*. (*b*) In both crystal structures rapid ${\text{Li}}^{+}$ *intra*cage dynamics is present which controls the $1/T_{1}$ ${\;}^{7}\text{Li}$ NMR rate peaks appearing near room temperature not only for X=Cl, Br but also for the poorly conducting compound with X=I (see *a*) [73]. For highly disordered ${\text{Li}}_{6}{\text{PS}}_{5}\text{Br}$ localized jump processes are characterized by $1/T_{1}$ NMR activation energies of 0.105 eV to 0.081 eV, which are even smaller than those seen for ${\text{Li}}_{6}{\text{PS}}_{5}\text{Cl}$. Site disorder is also advantageous for long-range ion transport as it results in lower high-*T* slopes of the spin-lock $1/T_{1\varrho }(1/T)$ NMR rate peaks for ${\text{Li}}_{6}{\text{PS}}_{5}\text{Br}$ (0.22 eV (X=Br) versus 0.27 eV (X=Cl)). (*c*) Controlled by both translational and rotational jump processes ${\;}^{31}\text{P}\;1/T_{1}$ reveals two SLR NMR rate peaks; most likely, the peak appearing at $T_{\text{max}}=220\;\text{K}$ mirrors rotational ${\text{PS}}_{4}^{3-}$ jumps that are dynamically decoupled from ${\text{Li}}^{+}$ diffusivity seen at higher *T* [74]. (Online version in colour.)

Meanwhile, the argyrodite-type thiophosphates ${\text{Li}}_{6}{\text{PS}}_{5}\text{X}$ (X=Cl, Br, I) belong to the most prominent examples that use anion disorder to manipulate cation (${\text{Li}}^{+}$) dynamics (and Li disorder) [31]. Anion substitution enhances ${\text{Li}}^{+}$ diffusivity, and similar effects were observed for glassy structures (mixed anion effect). While in ${\text{Li}}_{6}{\text{PS}}_{5}\text{I}$ the anions ${\text{I}}^{-}$ and ${\text{S}}^{2-}$ occupy distinct crystallographic positions regularly, in ${\text{Li}}_{6}{\text{PS}}_{5}\text{Br}$ strong anion site disorder is present and ${\text{Br}}^{-}$ and ${\text{S}}^{2-}$ are distributed over the sites 4*a* and 4*d*, see figure 9. It turned out that anion disorder, which is also seen to a lesser extent for ${\text{Li}}_{6}{\text{PS}}_{5}\text{Cl}$, is not the only origin to explain the high ${\text{Li}}^{+}$ self-diffusivity in ${\text{Li}}_{6}{\text{PS}}_{5}\text{Br}$; recent neutron diffraction measurements [75] show that the Li-ions do also occupy several interstitial sites in the argyrodite framework generating multiple hopping pathways, see figure 9*a*. In general, this situation resembles that of many mobile charge carriers being placed on a disordered lattice [76].

The ${\;}^{7}\text{Li}$ NMR responses of both ${\text{Li}}_{6}{\text{PS}}_{5}\text{Br}$ and ${\text{Li}}_{6}{\text{PS}}_{5}\text{Cl}$ are shown in the middle of figure 9. Two well-resolved SLR NMR rate peaks are seen near room temperature reflecting extremely fast ion exchange processes with residence times on the nanosecond time scale at $T_{\text{max}}$ and above. In agreement with the extent of structural site disorder, the activation energy derived from NMR is considerably lower for ${\text{Li}}_{6}{\text{PS}}_{5}\text{Br}$ (0.105 eV) as compared to that extracted for ${\text{Li}}_{6}{\text{PS}}_{5}\text{Cl}$ from the low-T NMR flank (0.165 eV) [73].

Importantly, the same rate peak is also seen for ${\text{Li}}_{6}{\text{PS}}_{5}\text{I}$ whose ionic conductivity is several orders of magnitude lower than those of the other two halides. Hence, local ${\text{Li}}^{+}$ hopping processes, which do not necessarily lead to long-range ion transport, are sufficient to generate this NMR response [73]. Most likely, *intra*cage ${\text{Li}}^{+}$ exchange is responsible for the NMR peak seen independently of the type of anion X. As suggested by both the cage-like structure and theory, concerted ion movements govern these rapid exchange processes [77], possibly also in the oxide analogues [78]. The prominent spin-lock NMR rate peaks of ${\text{Li}}_{6}{\text{PS}}_{5}\text{Br}$ and ${\text{Li}}_{6}{\text{PS}}_{5}\text{Cl}$ reflect long-range ion dynamics also involving *inter*cage Li hopping. Again, much lower activation energies are probed for ${\text{Li}}_{6}{\text{PS}}_{5}\text{Br}$ that benefits from stronger site disorder [75], polarisability effects [31] and, presumably, also from on-resonant rotational dynamics of the ${\text{PS}}_{4}^{3-}$ dynamics that trigger translational ion transport [74]. As has been shown recently, for X = Br the rotational motional correlation rate sensed by ${\;}^{31}\text{P}$ SLR NMR is close to that of the Li motional correlation rate [74]. Even faster rotational dynamics are seen for the ordered ${\text{Li}}_{6}{\text{PS}}_{5}\text{I}$ compound with its larger lattice constant [74]. The missing disorder and additional free space seems to be beneficial for polyanion dynamics. In figure 9, the ${\;}^{31}\text{P}$ SLR NMR rate passes though two rate maxima; while the one at lower T might mirror rotational ion dynamics, the peak at higher T appears at the same $T_{\text{max}}$ as the ${\;}^{7}\text{Li}$ SLR NMR rate peak originating from *intra*cage translational dynamics. Hence, the ${\;}^{31}\text{P}$ NMR spins indirectly sense the spin-fluctuations originating from the jumping Li cations.

### (d) Guiding the ions by low dimensional diffusion pathways

**Figure 10.**
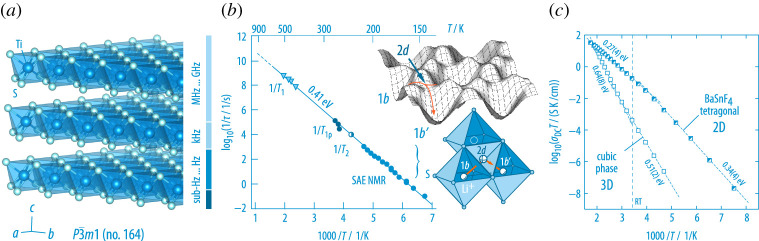
(*a*) The layered crystal structure of $1{\text{T-TiS}}_{2}$; guest species can reversibly populate the available voids in the van-der-Waals gap. (*b*) Arrhenius plot of the ${\text{Li}}^{+}$ jump rates as derived by the different NMR methods used to probe cation jumps [32,79]. The inset illustrates the potential landscape of octahedral and tetrahedral voids in isostructural ${\text{Li}}_{x}{\text{TiSe}}_{2}$ [80]; the jump pathway involving the tetrahedral void acting as intermediate position for the mobile Li-ions is shown below. (*c*) Change of the ionic conductivity of ${\text{BaSnF}}_{4}$ when going from the cubic form to the layer-structured modification [81]. In layered ${\text{BaSnF}}_{4}$ extremely rapid F anions result in a room-temperature ion conductivity of $0.7\;{\text{mS cm}}^{-1}$. (Online version in colour.)

On the one hand, spatial confinements may sensitively restrict the mobility of small cations in crystalline matter. On the other hand, many layer-structured materials offering, for example, an appropriate van der Waals space for guest species (figure 10), are extremely fast ion conductors making them highly powerful battery materials (${\text{LiCoO}}_{2}$-based active materials, graphite and graphite-based anode materials).

Layer-structured $1{\text{T-TiS}}_{2}$, see figure 10, used in one of the first Li-based batteries developed by Whittingham [82], belongs to the most intensively studied examples whose dynamic properties have also been investigated by ${\;}^{7}\text{Li}$ NMR [79]. It can accommodate Li-ions up to the composition ${\text{Li}}_{x=1}{\text{TiS}}_{2}$. Its crystal structure is depicted in figure 10. The Li-ions occupy the octahedral sites directly inside the van der Waals gap. At low levels of x≈0.7, ${\;}^{7}\text{Li}$ spin-alignment echo NMR spectroscopy [32,79] revealed that jumping from site to site occurs via the face-sharing tetrahedral sites as the octahedral sites are interconnected only via common edges hindering ion diffusion. The corresponding potential landscape, which was found for the isostructural system ${\text{TiSe}}_{2}$ [80], and the microscopic jump diffusion pathway are shown in the insets of figure 10. Altogether, the various NMR techniques were able to trace a fast and single diffusion pathway over a dynamic range of *ca* 10 orders of magnitude [83]. Furthermore, its two-dimensional nature has been confirmed by frequency-dependent SLR NMR measurements [83,84]. The structural basis of Li-ion diffusion in nanocrystalline and amorphous ${\text{Li}}_{x}{\text{TiS}}_{2}$ was studied in detail by Winter and Heitjans [85,86].

**Figure 11.**
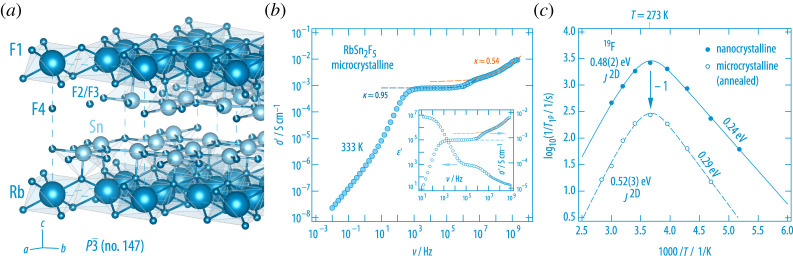
(*a*) Crystal structure of ${\text{RbSn}}_{2}{\text{F}}_{5}$ offering rapid F anion exchange and activation energies of 0.5 eV as probed by variable-temperature conductivity measurements. (*b*) Full conductivity isotherm of polycrystalline ${\text{RbSn}}_{2}{\text{F}}_{5}$ (298 K) recorded from the sub-Hz to the GHz range [87]. The isotherm is composed of two DC plateaus which we assign to the grain-boundary response and the bulk response. A Jonscher exponent of κ≈0.5 indicates two-dimensional ionic conduction. The inset compares the isotherm with the permittivity response of the ternary fluoride. (*c*) Purely diffusion-induced ${\;}^{19}\text{F}$ NMR spin-lock rates of micro- and nanocrystalline ${\text{RbSn}}_{2}{\text{F}}_{5}$ [87]. In both cases, short-range motions are to be characterized by activation energies lower than 0.3 eV. The full response is only consistent with results from conductivity spectroscopy if a spectral density function *J* developed for two-dimensional diffusion is used to parameterize the Lorentzian-shaped, but asymmetric rate peak(s). The two-dimensional spectral density function of Richards (see solid and dashed lines) contains a logarithmic frequency dependence of the rate $1/T_{1}$ in the high-*T* limit. (Online version in colour.)

${\text{BaSnF}}_{4}$ is another famous two-dimensional ion conductor. Whereas its cubic counterpart offers three-dimensional ionic conduction at a relatively high activation energy (figure 10), anion dynamics in the layer-structured counterpart is governed by values as low as 0.27 eV [81]. At room temperature, the DC conductivity $\sigma_{\text{DC}}$ almost reaches the $1\;{\text{mS cm}}^{-1}$ threshold. Replacing ${\text{Ba}}^{2+}$ partly by ${\text{Rb}}^{+}$ yields even higher values. Thus, together with ${\text{PbSnF}}_{4}$ it definitely belongs to the fastest F anion conductors known so far. Layer-structured ${\text{RbSn}}_{2}{\text{F}}_{5}$ (figure 11) joins this class of materials; it can be prepared by mechanochemical reaction of the binary fluorides and subsequent soft annealing [87].

Already the room-temperature conductivity isotherm of ${\text{RbSn}}_{2}{\text{F}}_{5}$ points to low-dimensional ion dynamics, see figure 11 [87]. While the low-frequency part of the isotherm is chiefly determined by electrode polarization, two distinct DC plateaus are visible at higher frequencies. The complete bulk response is only visible if measurements up to the GHz range are performed. The bulk (DC) plateau merges into a dispersive regime that follows a Jonscher power law ($\propto \nu^{\kappa }$) with the exponent κ being close to 0.5. Such a value is expected for two-dimensional ionic conduction. Indeed, the diffusion-controlled $1/T_{1\varrho }(1/T)$ ${\;}^{19}\text{F}$ NMR rate measurements of nano- and microcrystalline ${\text{RbSn}}_{2}{\text{F}}_{5}$ are only consistent with results from the conductivity measurements if they are analysed in terms of the semi-empirical expression for two-dimensional spin fluctuation as introduced by Richards [87]. Only if we assume two-dimensional ionic conduction, the NMR response yields an activation energy for long-range ion transport of *ca* 0.5 eV, which is in agreement with $E_{a}$ as probed by $\sigma_{\text{DC}}$. Local jump processes, to which the low-T flank of the $1/T_{1\varrho }(1/T)$ ${\;}^{19}\text{F}$ NMR peak is sensitive, are characterized by activation energies in the order of 0.25 eV. Again, the asymmetry of the rate peaks points to correlated ion dynamics governing overall transport in the ternary fluoride.

Quite recently, fast ${\text{Li}}^{+}$ ion transport has also been found for layer-structured hectorite ${\text{Li}}_{0.5}[{\text{Mg}}_{2.5}{\text{Li}}_{0.5}]{\text{Si}}_{4}{\text{O}}_{10}{\text{F}}_{2}$ being the first silicate showing high bulk ion conductivities in the order of $0.1\;{\text{mS cm}}^{-1}$ (0.35 eV) [88]. Although hardly affecting ion conductivity in ${\text{RbSn}}_{2}{\text{F}}_{5}$, grain boundaries may, in general, block fast intragrain ion dynamics [53,89]. In contrast to the mechanically softer fluorides and sulfides, for the hectorite-type silicate this blocking effect impedes facile long-range transport over larger distances. Suitable interfacial engineering, see below, is thus necessary to liberate its full potential as fast ion conductor.

Further materials with two-dimensional Li-ion diffusion [90], which have been studied by variable-temperature NMR relaxometry, include ${\text{LiBH}}_{4}$ [33], ${\text{Li}}_{x}{\text{NbS}}_{2}$ [91], ${\text{Li}}_{x}{\text{SnS}}_{2}$ [90,92], for example. Features being consistent with fast one-dimensional diffusion were found for $\beta {\text{-Li}}_{3}{\text{PS}}_{4}$ [93] and ${\text{Li}}_{12}{\text{Si}}_{7}$ [34]; in some cases, the presence of low-dimensional ion dynamics was unequivocally probed by frequency-dependent NMR SLR measurements [90]. Quite recently, the metal organic framework MIL-121 was functionalized such that fast Li and Na conduction occurs along the inner channels of the non-conducting host [94]. For the Na-MIL-121, ionic conductivities in the order of $0.1\;{\text{mS cm}}^{-1}$ were reached at room temperature. This example, taking benefit from *inner*facial cation pathways, guides us to the last class of materials that exhibit (structurally disordered) *inter*facial regions (IRs). Only in nanocrystalline materials is the volume fraction of such regions high enough to host a sufficiently large number of spins to be detectable by NMR. As mentioned above, prominent single-phase examples include binary fluorides such as high-energy ball milled ${\text{BaF}}_{2}$ [61] and the oxides ${\text{LiMO}}_{3}$ (M=Nb, Ta) [30,95] and ${\text{LiAlO}}_{2}$ [96] as well as glass forming borates and silicates [29]. In some cases, NMR is also able to differentiate between the less mobile spins residing in the grains and those in or near the IRs, see the SLR NMR and lineshape studies on ${\text{LiBH}}_{4}$ [35], ${\text{LiNbO}}_{3}$ [95], ${\text{Li}}_{2}\text{O}$ [36] and on the related composites ${\text{Li}}_{2}\text{O}:{\text{Al}}_{2}{\text{O}}_{3}$ and ${\text{LiF : Al}}_{2}{\text{O}}_{3}$ [40,97]. An overview and an introduction into this topic is given elsewhere [98].

**Figure 12.**
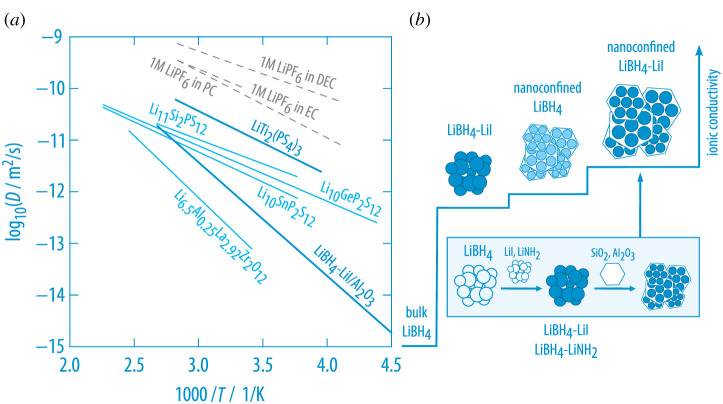
(*a*) Diffusion coefficients of nanoconfined $({\text{LiBH}}_{4}/\text{LiI}):{\text{Al}}_{2}{\text{O}}_{3}$ as derived from pulsed field gradient (PFG) NMR, NMR relaxometry and conductivity spectroscopy [100]. The values are compared with those of LTPS, which were also determined by PFG NMR. The two Arrhenius lines indicate that very similar diffusion coefficients are obtained at *ca* 400 K due to the higher Arrhenius pre-factor of the nanoconfined composite. For comparison, diffusion coefficients from other solid and liquid electrolytes (see dashed lines) are included as well [50]. (*b*) Increase of the ionic conductivity when going from bulk ${\text{LiBH}}_{4}$ to the LiI-stabilized form and further to the nanoconfined composite material; a similar trend is also seen for ${\text{LiNH}}_{2}:\text{LiI}$ in contact with ${\text{SiO}}_{2}$ or ${\text{Al}}_{2}{\text{O}}_{3}$ [99]. (Online version in colour.)

In the nanoconfined two-phase, dispersed ion conductor $({\text{LiBH}}_{4}/\text{LiI}):{\text{Al}}_{2}{\text{O}}_{3}$ fast ionic conduction is probed on different length scales by both conductivity spectroscopy and NMR [99,100]. Diffusion coefficients D as measured by the pulsed field gradient (PFG) NMR, being able to probe the macroscopic tracer diffusion coefficient, agree very well with those that were deduced from SLR NMR and conductivity spectroscopy (figure 12) [100]. Importantly, ${\text{LiBH}}_{4}$ is known to adopt two crystal structures; whereas the poorly conducting orthorhombic form is only stable at sufficiently low T, the hexagonal polymorph, exhibiting rapid translational Li-ion motions, is stable at higher T. Addition of LiI stabilizes the layered modification further (figure 12). Bulk ion dynamics in spatially confined (hexagonal) ${\text{LiBH}}_{4}/\text{LiI}$ needs, thus, to be described by a two-dimensional (d=2) transport mechanism, whereas the overall, macroscopic Li-ion transport in $\text{nano-}({\text{LiBH}}_{4}/{\text{LiNH}}_{2}):{\text{Al}}_{2}{\text{O}}_{3}$ turned out to be a three-dimensional process [100]. Most likely, the conductor:insulator interface provides a network of highly conducting pathways for the charge carriers. In such a (surface) percolation lattice [66] diffusion can be considered as taking place in a fractal system with the so-called Mandelbrot dimension $\bar{d}$ being $2<\bar{d}<3$ [101]. A similar trend of the ion conductivities as illustrated in figure 12*b* is also probed for $\text{nano-}({\text{LiNH}}_{2}/\text{LiI})$:insulator with either ${\text{Al}}_{2}{\text{O}}_{3}$ or ${\text{SiO}}_{2}$ acting as ionically insulating phase [99].

**Figure 13.**
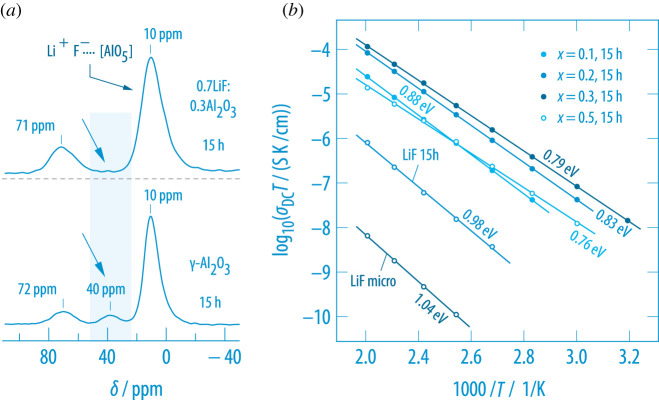
(*a*) ${\;}^{27}\text{Al}$ MAS NMR spectra of nanocrystalline ${\text{LiF:Al}}_{2}{\text{O}}_{3}$ revealing three magnetically inequivalent Al species for ${\text{nano-Al}}_{2}{\text{O}}_{3}$ that crystallizes with the γ form [97]. F anions saturate the unsaturated, penta-coordinated Al species near the surface regions. (*b*) Change of the ionic conductivity of $(1-x)\text{LiF}:x{\text{Al}}_{2}{\text{O}}_{3}$ with increasing insulator content. At too high amounts of ${\text{Al}}_{2}{\text{O}}_{3}$ the percolating network gets interrupted and $\sigma_{DC}$ decreases again. For comparison, the change of ionic conductivity of unmilled LiF with temperature (1.04 eV) is also shown. (Online version in colour.)

In nanocrystalline ${\text{LiF:Al}}_{2}{\text{O}}_{3},$ this interface does also play a significant role in explaining the analogous enhancement [97]. Adding $\gamma {\text{-Al}}_{2}{\text{O}}_{3}$ to nano-LiF causes the ionic conductivity of poorly conducting nano-LiF to increase by several orders of magnitude, see figure 13. Starting with nano-LiF, the activation energy decreases from 0.98 eV to 0.79 eV if x in $(1-x)\text{LiF}:x{\text{Al}}_{2}{\text{O}}_{3}$ is increased from 0 to 0.3. To shed light on the microscopic origin of this effect, which has frequently been explained in the frame of the space charge model [102,103], we recorded ${\;}^{27}\text{Al}$ MAS NMR spectra to probe the direct environment of the Al species [97]. Besides the octa- (*ca* 10 ppm) and tretrahedrally coordinated Al species (*ca* 70 ppm) of the bulk regions, in ball-milled $\gamma {\text{-Al}}_{2}{\text{O}}_{3}$ a non-negligible amount of Al resides in the interfacial regions. These non-saturated Al centres appear as penta-coordinated species in NMR. Adding LiF to $\gamma {\text{-Al}}_{2}{\text{O}}_{3}$ being equipped with penta-Al near the surface regions yields an NMR spectrum whose penta-Al signal vanishes. This change can be explained by forming ${\text{AlO}}_{5}\text{F}$ species whose isotropic NMR signal appears close to δ=10 ppm. The formation of saturated Al centres according to $[{\text{AlO}}_{5}]\cdots {\text{F}}^{-}-{\text{Li}}^{+}$ might change the interfacial regions by creating ${\text{Li}}^{+}$ vacancies. Such defect-rich regions, presumably being the microscopic origin of extended space charge zones [41,102,103], are expected to easily transport Li-ions on a long-range length scale. Further interfacial engineering of these regions provides another degree of freedom to successfully manipulate three-dimensional ion dynamics in nanocrystalline ceramics.

## 3. Conclusion and summary

Understanding the circumstances that lead to fast ion transport in ceramics is a vital research topic. We presented a selection of model compounds, including also materials inspired by applications, whose ion dynamics were studied by diffusion-induced time-domain NMR methods. SLR NMR, as it is widely applied in our group to study the elementary steps of ion hopping, does not only give access to energy barriers determining local barriers but also provides insights in long-range ion transport. In particular, the latter information is included in NMR stimulated (or spin-alignment) echoes and in the high-T flanks of the SLR NMR rate peaks. In favourable cases, high-resolution techniques can be used to directly visualize the preferred hopping motions as was exemplarily shown for the solid-solution ${\text{Pb}}_{1-x}{\text{Ca}}_{x}{\text{F}}_{2}$.

In general, to identify the origins of fast ion dynamics, information from NMR needs to be compared to and complemented with results from other microscopic or macroscopic methods and vice versa. Further methods, either being nuclear or non-nuclear in nature, include AC conductivity spectroscopy [104,105], tracer experiments (radio tracer, PFG NMR) [106–108] and even less popular methods such as beta-NMR [109,110] and muon spin resonance [111]. As an example, the latter technique has recently been applied, together with NMR, to study ion dynamics and the associated diffusion pathways in spinel-type ${\text{Li}}_{2}{\text{NiGe}}_{3}{\text{O}}_{8}$ [111].

In many cases, each method has its own metric and senses a particular aspect of diffusive motion on a specific time and length scale. Hence, it is important, in the sense of a holistic approach, to monitor the change and the shape of the underlying motional correlation functions preferable over a large dynamic range. Of course, the proper interpretation of experimental results needs both highly accurate structural data and input from theoretical calculations. Then, in some cases, it is possible to identify the main origins of rapid self-diffusion. Here, apart from the right choice of polyhedra connections, bonding situations and the correct size of the diffusion pathways, we differentiated the influencing variables roughly into five classes. Under the umbrella of macroscopic or microscopic (site) disorder, to which also geometric frustration belongs, we see that (1) partially occupied sublattices (${\text{Li}}_{4+x}{\text{Ti}}_{5}{\text{O}}_{12}$, ${\text{Li}}_{7}{\text{La}}_{3}{\text{Zr}}_{2}{\text{O}}_{12}$) as well as lattice strain and polyhedra deformations play important roles in changing local (electronic and magnetic) structures to enhance ion dynamics. Strain and deformations are the result of (2) mixing cation or anions differing in size and charge $\left((\text{Me},\text{Ca}){\text{F}}_{2}\;(\text{Me}=\text{Pb},\;\text{Ba}),\;{\text{Li}}_{6}{\text{PS}}_{5}\text{X}\;(\text{X}=\text{Br},\;\text{Cl},\;\text{I})\right)$. High-energy ball milling acts as a suitable tool to force the formation of such non-equilibrium compounds characterized by a mismatch in cation size. In many cases, these materials are to be characterized by strongly heterogeneous potential landscapes rather than being periodically uniform. If these landscapes do also provide interconnected regions with flat energy potentials, as is also seen for the unique structure of ${\text{LiTi}}_{2}{\left({\text{PS}}_{4}\right)}_{3}$, any site preference gets lost, as is the case for the diffusion of many particles on disordered lattices [76]. Here, this extremely fast charge carrier hopping is expected not only on a local length scale. Of course, any reduction in activation energy should not be compensated by a decrease in attempt frequency.

Moreover, we have seen that translational ion dynamics may be synergistically coupled with (3) rotational dynamics of polyanions (e.g. boron hydrides, thiophosphates). Altering the polyanion framework by substitution effects will not only change rotational or librational motions but simultaneously influence the bottlenecks of the translational diffusion pathways.

In particular, (4) in many low-dimensional (two-dimensional) materials (layered dichalcogenides, see ${\text{Li}}_{x}{\text{TiS}}_{2}$) and fluorides such as ${\text{MeSnF}}_{4}$ (Me=Pb, Ba) or ${\text{RbSn}}_{2}{\text{F}}_{5}$ we observe fast ion transport along their buried interfaces that guide the ions along a spatially extended pathway. On the more macroscopic scale, (5) surface and interfacial regions of nanocrystalline materials, which again take advantage of structural disorder and space charge effects especially introduced or intensified by insulating phases (e.g. ${\text{LiBH}}_{4}:{\text{Al}}_{2}{\text{O}}_{3}$, ${\text{LiF:Al}}_{2}{\text{O}}_{3}$), can be manipulated to control ionic transport across the whole sample.

Without any doubt, it remains a challenge to identify even more factors and to refine the picture as it is seen by NMR. Further work needs suitable, if not unique, model systems to study the interplay of these influencing factors. It is clear that compromises have to be made if we include demands from application-driven research. Independent of the scientific discipline, it is quite generally accepted that there is a long way from basic research to market-ready solutions.

## Data accessibility

This article has no additional data.
